# Optimization of the enrichment medium for recombinant *ChIL-4-ChIL-2* in *Lactococcus lactis* through response surface methodology

**DOI:** 10.1016/j.psj.2025.105025

**Published:** 2025-03-14

**Authors:** Hao-yu Zhang, Yu-tong Yang, Xue-qi Zhang, Si-chen Dong, Li-wen Wang, Ying-ying Chen, Li Zhang, Xiao-ling Lv, Rui Bai, Ming-xue Zheng

**Affiliations:** College of Veterinary Medicine, Shanxi Agricultural University, Taigu, Jinzhong, 030801, China

**Keywords:** Coccidiosis live vaccine, Recombinant L.lactis NZ3900/pNZ8149-IL-4-IL-2, Box-Behnken design, Response surface methodology experiments

## Abstract

Coccidiosis, caused by Eimeria, represents a significant challenge to the poultry industry and leads to significant economic losses to the poultry industry. Traditional anticoccidial drugs is diminishing due to drug resistance, and although live vaccines are an effective preventive and treatment means, they may cause mild infections and affect weight gain when used. Recombinant *ChIL-4-ChIL-2* in *Lactococcus lactis*, as a new type of food-grade recombinant cytokine immunoadjuvant, characterized by its ability to enhance the immunological efficacy of live vaccines against chicken coccidiosis. In this study, using the OD_600_ value of the culture broth of *ChIL-4-ChIL-2* in *Lactococcus lactis* as the index, the optimal combination of carbon and nitrogen sources and inorganic salts in the medium was determined through single-factor experiments, and the medium formula was optimized using Box-Behnken design and response surface analysis. The results of single-factor experiments indicated that the optimal carbon source, nitrogen source, and buffer salt addition amounts in the M17 medium for recombinant *ChIL-4-ChIL-2 in Lactococcus lactis* were as follows: 1 % mixed carbon source (with a trehalose to glucose ratio of 1:1), 7 % yeast extract powder as the nitrogen source, and 0.5 % potassium dihydrogen phosphate-sodium hydroxide as the buffer salt. Furthermore, the results of response surface methodology experiments indicate that the optimal medium formulation for recombinant ChIL-4-ChIL-2 in *Lactococcus lactis* is M17 medium supplemented with 1 % carbon source (glucose: trehalose = 1:1), 8 % nitrogen source (yeast extract powder), and 0.8 % buffer salts (potassium dihydrogen phosphate-sodium hydroxide). This optimized medium can significantly increase the yield of recombinant *ChIL-4-ChIL-2* in *Lactococcus lactis*, and the OD_600_ value of the cell density can reach 1.103. This optimized medium provides a scientific foundation for developing a production process aimed at the large-scale production of recombinant *ChIL-4-ChIL-2* in *Lactococcus lactis*.

## Introduction

Chicken coccidiosis is an intestinal parasitic disease caused by the infection of Eimeria in chickens, characterized by intestinal damage, diarrhea, or bloody stools(Ahmad et al., 2024; Blake et al., 2020). Currently, the annual loss caused by chicken coccidiosis infection worldwide can reach approximately 10.4 billion pounds (about 88 billion yuan). Long-term or improper use of the same anticoccidial drug will lead to drug resistance in coccidia, and after the infected chickens show bloody stools, the existing coccidiostats have almost no effect on the reproduction and damage of chicken coccidia(Attree et al., 2021). At present, live vaccines are the only effective way to prevent and control of chicken coccidiosis. They can stimulate the natural immunity of chickens, reduce drug resistance, improve production performance, and avoid drug residues. However, live vaccines still have defects such as slow generation of immune protection, long immune protection gap period, and affecting the weight gain of chicks(Zheng et al., 2023).

To this end, our team has successfully constructed a new type of food-grade recombinant *ChIL-4-ChIL-2* in *Lactococcus lactis* without drug resistance genes, with higher safety standards, reduced production costs, and facilitating oral administration. The adjuvant can enhance the humoral and cellular immune responses, promote mucosal immunity, and improve the immune protection effect of live vaccines against chicken coccidiosis from moderate to high efficiency. It also has the function of regulating the intestinal flora. However, there are still problems in the culture process, such as expensive medium and low culture density, which make it impossible to achieve large-scale culture.

For veterinary biological products, an efficient, low-cost, and easy-to-scale-up production process must be established when entering the clinical and production research stage from the laboratory. In this study, recombinant *ChIL-4-ChIL-2* in *Lactococcus lactis* was used as the research object, and its culture broth OD_600_ value was used as the primary detection index. The optimal enrichment medium for recombinant *ChIL-4-ChIL-2* in *Lactococcus lactis* was determined using single-factor experiments and response surface methodology. Finally, the optimal medium formula for the recombinant strain was optimized to provide a basis and necessary conditions for the subsequent large-scale production of recombinant *ChIL-4-ChIL-2* in *Lactococcus lactis*.

## Materials and methods

### Materials

#### Strain source

Recombinant *ChIL-4-ChIL-2* in *Lactococcus lactis* (L*. lactis* NZ3900/pNZ8149-*ChIL-4-ChIL-2*, abbreviated as RLIL4/2) was prepared and stored by the Veterinary Pathology Laboratory at the College of Veterinary Medicine, Shanxi Agricultural University.

#### Main reagents and instruments

D-anhydrous glucose (Beijing Solarbio Science & Technology Co., Ltd., Lot.NO: 616R022), glycerol (G-CLONE, Lot.NO: 112A031), trehalose (G-CLONE, Lot.NO: 0111A0201), sodium glutamate (G-CLONE, Lot.NO: 131A0201), sodium ascorbate (G-CLONE, Lot.NO: 513A0201), skim milk powder (G-CLONE, Lot.NO: 301A0412), soybean peptone (Qingdao Hope, Lot.NO: 20230515), peptone (Beijing Solarbio Science & Technology Co., Ltd., Lot.NO: 929M053), tryptone (Qingdao Hope, Lot.NO: 20230925), yeast extract powder (Qingdao Hope, Lot.NO: 20230624), beef extract (Beijing Solarbio Science & Technology Co., Ltd., Lot.NO: 920P051), lactose (Beijing Solarbio Science & Technology Co., Ltd., Lot.NO: 705I021), sucrose (Sinopharm Chemical Reagent Co., Ltd., Lot.NO: 20230514), β-glycerophosphate sodium (Macklin, Lot.NO: C13957268), clean bench (Suzhou Purification: SW-CJ-1F), full-wavelength microplate reader (Thermo Fisher: 1530), autoclave (HIRAYAMA: HVE-50), CO_2_ incubator (Thermo Fisher Scientific, USA).

#### Preparation of M17 Medium

The M17 medium was prepared according to the method of Terzaghi(Terzaghi and Sandine, 1975). Weigh 0.5 g of soybean peptone, 0.2 g of peptone, 0.25 g of casein peptone, 0.25 g of yeast extract powder, 0.5 g of beef extract, 0.5 g of lactose, 0.05 g of sodium ascorbate, 1.9 g of β-glycerophosphate sodium, and 0.025 g of magnesium sulfate, add 100 mL of deionized water and mix well, adjust the pH to 7, and autoclave at 115°C for 15 min.

### Methods

#### Strain cultivation

The frozen recombinant *ChIL-4-ChIL-2* in *Lactococcus lactis* was streaked onto a GM17 solid culture plate and incubated at 30°C for 24 h for resuscitation. A single colony was picked and inoculated into 5 mL of GM17 liquid medium and incubated overnight at 30°C to serve as the seed culture.

#### Single-factor experimental screening

(1)Carbon Source Screening

The carbon-free M17 liquid medium (No carbon source components are added to the M17 medium) was divided into 4 groups, and 0.5 % glucose, lactose, sucrose, and trehalose were added as different carbon sources, respectively. At the same time, a group consisting of M17 medium was set as a control. There were 3 Erlenmeyer flasks in each group, with 10 mL of medium in each flask. 5 % of the seed culture was added to each flask, and the flasks were incubated statically at 30°C for 8 h. 200 μL of culture broth was taken from each Erlenmeyer flask, and the OD_600_ value was measured with the uninoculated culture broth as a blank for zero adjustment. The carbon source with the highest OD_600_ value in the culture broth was selected as the optimal carbon source for the medium.

(2)Nitrogen Source Screening

The nitrogen-free GM17 liquid medium (No nitrogen source components are added to the M17 medium) was divided into 7 groups, and 1.75 % soybean peptone, peptone, casein peptone, tryptone, yeast extract powder, and beef extract were added as different nitrogen sources, respectively. At the same time, a group of composite nitrogen source GM17 was set as a control. There were 3 Erlenmeyer flasks in each group, with 10 mL of medium in each flask. 5 % of the seed culture was added to each flask, and the flasks were incubated statically at 30°C for 8 h. The OD_600_ value was measured, and the nitrogen source with the highest OD_600_ value in the culture broth was selected as the optimal nitrogen source for the medium.

(3) Buffer Salt Screening

The buffer salt-free M17 liquid medium (The M17 medium does not contain buffer salt components) was divided into 3 groups, and 2 % β-glycerophosphate sodium and potassium dihydrogen phosphate - sodium hydroxide were added as different buffer salts, respectively. The medium without buffer salt was used as the control group. There were 3 Erlenmeyer flasks in each group, with 10 mL of medium in each flask. 5 % of the seed culture was added to each flask, and the flasks were incubated statically at 30°C for 8 h. The OD_600_ value was subsequently measured, and the buffer salt with the highest OD_600_ value in the culture broth was selected as the buffer salt for the medium.

#### Optimization of the proportions of carbon and nitrogen sources and buffer salts in the enrichment medium for recombinant *ChIL-4-ChIL-2* in *Lactococcus lactis*

(1)Optimization of the Carbon Source in the Enrichment Medium

The carbon-free M17 medium was divided into 7 groups, with 3 tubes in each group and 8 mL of medium in each tube. Carbon sources composed of 0.5 % trehalose and glucose in ratios of 1:1, 1:2, 1:3, 3:1, and 2:1 were added, respectively, and trehalose (T) and glucose (G) were used as single carbon source control groups. Each group of media was inoculated with 0.4 mL of seed culture and incubated statically at 30°C for 8 h. 200 μL of the culture broth was extracted from each tube, and the OD_600_ value was measured with the medium as a blank for zero adjustment.

The M17 medium was divided into 5 groups, with 3 tubes in each group and 8 mL of medium in each tube. Various concentrations of the optimal ratio of trehalose and glucose composite carbon source (0.0 %, 0.25 %, 0.50 %, 1.0 %, and 1.5 %) were added to each group. The methods for culture and measurement were consistent with those previously described.

(2)Optimization of the Nitrogen Source in the Enrichment Medium

The carbon-free and nitrogen-free M17 medium was divided into 8 groups, with 3 tubes in each group and 8 mL of medium in each tube. 0.5 % glucose was added as the carbon source, and 1 %, 2 %, 3 %, 4 %, 5 %, 6 %, 7 %, and 8 % yeast extract powder was added as the nitrogen source, respectively. Each group of media was inoculated with 0.4 mL of seed culture and incubated statically at 30°C for 8 h. 200 μL of the culture broth was extracted from each tube, and the OD_600_ value was measured with the medium as a blank for zero adjustment.

(3)Optimization of the Buffer Salt in the Enrichment Medium

The buffer salt-free GM17 medium was divided into 5 groups, with 3 tubes in each group and 8 mL of medium in each tube. Various concentrations of potassium dihydrogen phosphate - sodium hydroxide (0.5 %, 1 %, 2 %, 2.5 %, and 3 %) were added as buffer salts. Each group of media was inoculated with 0.4 mL of seed culture and incubated statically at 30°C for 8 h, and the OD_600_ value was measured.

#### Response surface optimization of the enrichment medium for recombinant *ChIL-4-ChIL-2* in *Lactococcus lactis*

Based on the results of the single-factor experiments, a Box-Behnken central composite experimental design was implemented. Three factors, namely the percentages of carbon source, nitrogen source, and buffer salt, were used as independent variables, while the OD_600_ value was used as the response variable for the design of the response surface experiment. The coding of factors and levels is presented in Table 1.

**Table 1:** Response Surface Test Factor Levels.

Level	Carbon source (%)	Nitrogen source (%)	Buffer salt (%)
−1	0.5	6.0	0.25
0	1.0	7.0	0.50
1	1.5	8.0	1.00

The nitrogen source and buffer salt for all experimental groups were added to the M17 medium, which was devoid of carbon, nitrogen, and buffer salts, in accordance with the formulation outlined in Table 2. The medium was then autoclaved at 115°C for 15 min, and subsequently cultured in erlenmeyer flasks. Each group consisted of three flasks, each containing 10 mL of the medium. The flasks were incubated statically at 30°C and the OD_600_ value was measured after 8 h. Following the experiment, the results were represented as response surface and contour plots, and the optimization results of the medium were compared and summarized.

**Table 2:** Box-Behnken experimental design.

Run	Carbon source		Nitrogen source		Buffer salt	
	Horizontal code	Add (%)	Horizontal code	Add (%)	Horizontal code	Add (%)
1	1	1.5	0	7.0	−1	0.25
2	1	1.5	0	7.0	1	1.00
3	0	1.0	0	7.0	0	0.50
4	1	1.5	1	8.0	0	0.50
5	1	1.5	−1	6.0	0	0.50
6	−1	0.5	0	7.0	−1	0.25
7	−1	0.5	−1	6.0	0	0.50
8	0	1.0	−1	6.0	−1	0.25
9	0	1.0	1	8.0	1	1.00
10	0	1.0	−1	6.0	1	1.00
11	0	1.0	0	7.0	0	0.50
12	0	1.0	0	7.0	0	0.50
13	−1	0.5	0	7.0	1	1.00
14	−1	0.5	1	8.0	0	0.50
15	0	1.0	1	8.0	−1	0.25

#### Verification experiment of recombinant *ChIL-4-ChIL-2* in *Lactococcus lactis*

The optimized medium formula obtained by the response surface model involved the incorporation of carbon sources, nitrogen sources, and buffer salts into a carbon-, nitrogen-, and buffer salt-free M17 medium at the optimized proportions and autoclaved at 115°C for 15 min. Erlenmeyer flask culture experiments were conducted, with each experimental group being replicated three times, with 10 mL of medium in each flask. The flasks were incubated statically at 30°C, and the OD_600_ value was measured after 8 h. The experimental results were then compared to the predicted values, and the relative error was calculated, with the acceptable error threshold set at ≤5 %.

### Data analysis

The experimental data were analyzed by one-way ANOVA using SPSS Statistics 29.0 statistical software. *p* < 0.05 was used as the significance criterion. The experimental results of each group were expressed as "mean ± standard error" (Mean ± SEM), and Graphpad prism 9.5.1 was used for graphing. The response surface design was analyzed using the software Degisn-Expert 13.0.

## Results and analysis

### Single-factor experiments

#### Screening carbon sources

**Fig. 1:**
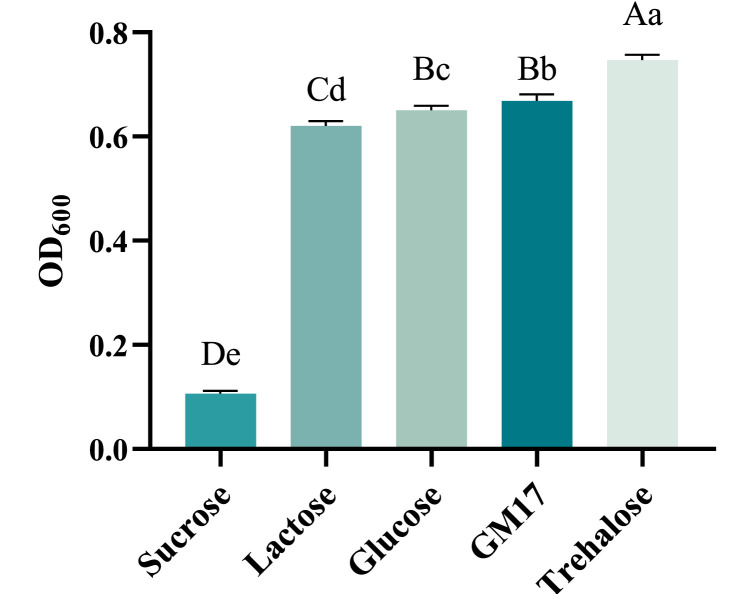
The influence of different carbon sources on the yield of RLIL4/2 strain. Note: Multiple comparisons were conducted utilizing the Duncan method. Distinct capital letters signify extremely significant differences between groups (*p* < 0.01), while distinct lowercase letters denote significant differences between groups (*p* < 0.05). This notation is also applicable to the subsequent figures.

The yields of recombinant *ChIL-4-ChIL-2* in *Lactococcus lactis* in M17 with trehalose, lactose combined with glucose (GM17), glucose, lactose, and sucrose, exhibited a decreasing trend. Notably, the cell yield obtained with trehalose was significantly higher than that achieved with the composite carbon source GM17(*p* < 0.01), as illustrated in Fig. 1.

#### Screening nitrogen sources

**Fig. 2:**
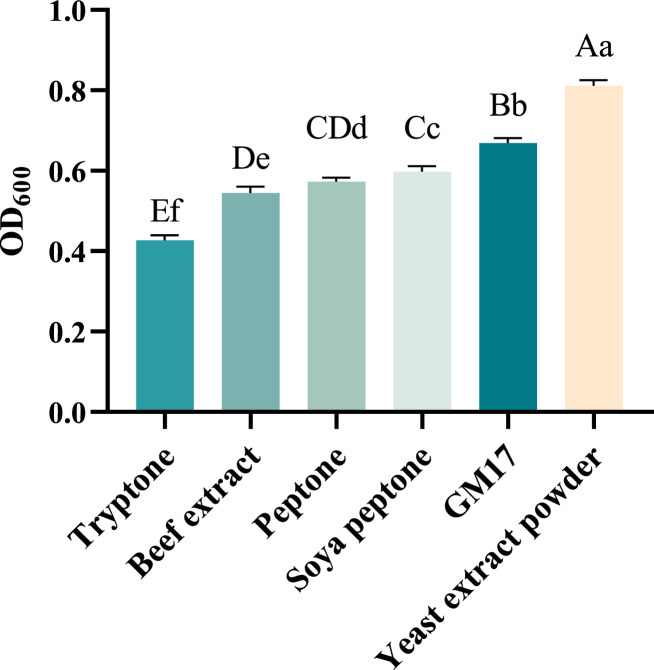
The influence of different nitrogen sources on the yield of RLIL4/2 strain.

As illustrated in Fig. 2, the yields of recombinant *ChIL-4-ChIL-2* in *Lactococcus lactis* in GM17 with various nitrogen sources—including yeast extract powder, composite nitrogen source (GM17), peptone, soybean peptone, beef extract, and tryptone—exhibited a decreasing trend. Notably, the yield of the recombinant strain in the yeast extract powder group was significantly higher (*p* < 0.05) compared to the yields observed in the other groups, as depicted in Fig. 2.

#### Screening of buffer salts

**Fig. 3:**
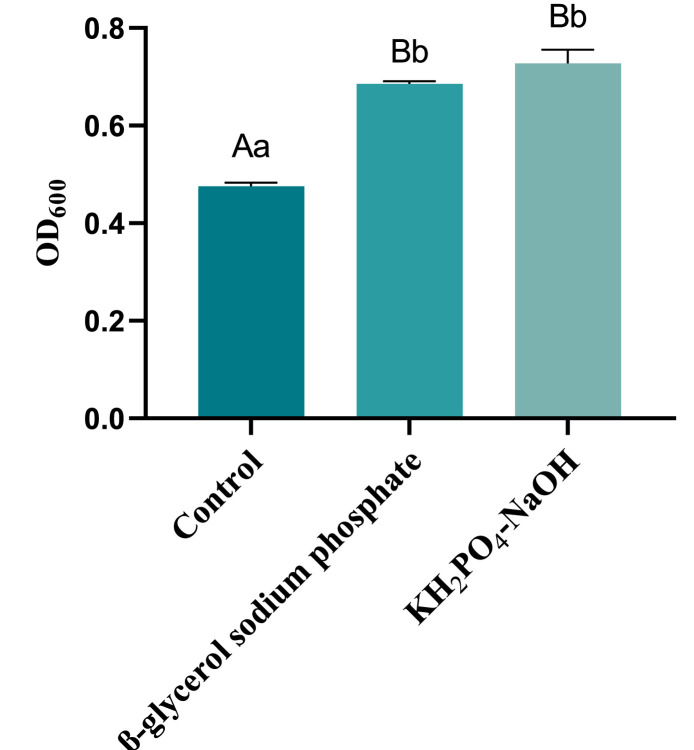
Growth of RLIL4/2 in M17 medium with different buffered salts.

The yields (OD_600_) of recombinant *ChIL-4-ChIL-2* in *Lactococcus lactis* in the M17 group supplemented with 2 % β-glycerophosphate sodium, as well as in the M17 group supplemented with potassium dihydrogen phosphate-sodium hydroxide, exhibited significantly higher yields (*p* < 0.05) compared to the M17 medium without supplementation. However, no significant difference (*p* > 0.05, *p* = 0.054) was observed between the yields in the β-glycerophosphate sodium M17 group and the potassium dihydrogen phosphate-sodium hydroxide M17 group, as illustrated in Fig. 3. Given that the cost of the β-glycerophosphate sodium M17 medium was higher than that of the potassium dihydrogen phosphate-sodium hydroxide M17 medium, the latter, being more economical, was selected as the buffer salt.

### Optimization of the proportions of carbon and nitrogen sources and buffer salts in the RLIL4/2 enrichment medium

#### Carbon source optimization

(1)Optimization of the Ratio of Composite Carbon Sources

**Fig. 4:**
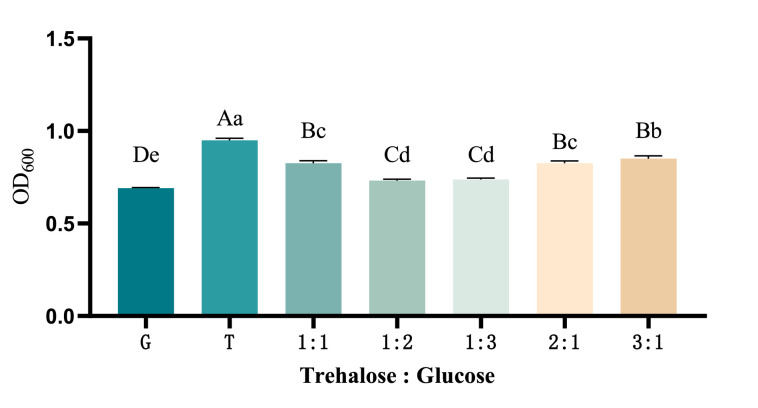
The impact of different carbon source ratios on the yield of RLIL4/2 strain.

The yields (OD_600_) of recombinant *ChIL-4-ChIL-2* in *Lactococcus lactis* was observed to decrease sequentially across groups utilizing trehalose (T), as well as combinations of trehalose and glucose in the ratios of 3:1, 2:1, 1:1, 1:3, and 1:2, in addition to a group using glucose (G) as the sole carbon source. Notably, the groups supplemented with trehalose exhibited significantly higher yields (*p* < 0.05) compared to the group that utilized only glucose (G). Furthermore, the yields (OD_600_) in the groups with trehalose and glucose at the ratios of 3:1, 2:1, and 1:1 were significantly greater (*p* < 0.05) than those in the groups with trehalose and glucose at the ratios of 1:3 and 1:2. To optimize cost-effectiveness, the 1:1 ratio was identified as the optimal carbon source ratio, as illustrated in Fig. 4.

(2)Optimization of the Carbon Source Concentration

**Fig. 5:**
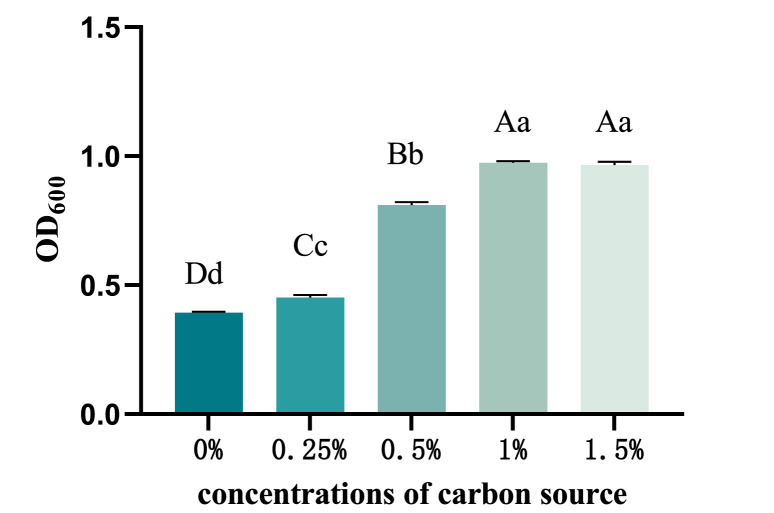
The impact of using different carbon source concentrations on the yield of RLIL4/2 strain.

Utilizing a 1:1 ratio of trehalose and glucose as the carbon source, the yields (OD_600_) of recombinant *ChIL-4-ChIL-2* in *Lactococcus lactis* exhibited a progressive increase across groups with carbon source concentrations of 0.0 %, 0.25 %, 0.50 %, 1.0 %, and 1.5 %. The yields (OD_600_) of the recombinant strains in the groups supplemented with carbon sources were significantly higher (*p* < 0.05) compared to those in the carbon-free M17 group (0.0 %). Furthermore, the OD_600_ values of recombinant *ChIL-4-ChIL-2* in *Lactococcus lactis* in the 1.5 % and 1.0 % carbon source groups were significantly elevated (*p* < 0.05) relative to those in the 0.0 %, 0.25 %, and 0.50 % carbon source groups. However, no significant difference (*p* > 0.05) was observed between the 1.5 % and 1.0 % carbon source groups, as illustrated in Fig. 5.

#### Nitrogen source optimization

**Fig. 6:**
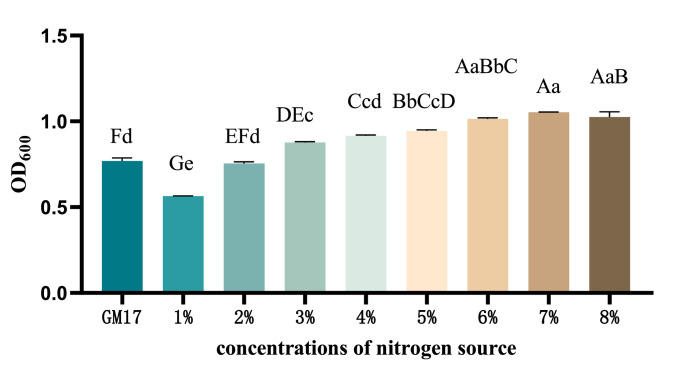
The effect of varying nitrogen source concentrations on the yield of RLIL4/2 strain.

Utilizing yeast extract powder as the sole nitrogen source, the yields (OD_600_) of recombinant *ChIL-4-ChIL-2* in *Lactococcus lactis* exhibited a progressive increase across groups with nitrogen source concentrations of 1 %, 2 %, 3 %, 4 %, 5 %, 6 %, 7 %, and 8 %. Notably, the OD_600_ values of the recombinant strains in groups supplemented with 3 %, 4 %, 5 %, 6 %, 7 %, and 8 % yeast extract powder were significantly greater (*p* < 0.05) than those observed in the GM17 control group. Furthermore, the yield (OD_600_) of the recombinant strain in the 7 % nitrogen source group was significantly higher (*p* < 0.05) compared to all other nitrogen source groups, as illustrated in Fig. 6.

#### Buffer salt optimization

**Fig. 7:**
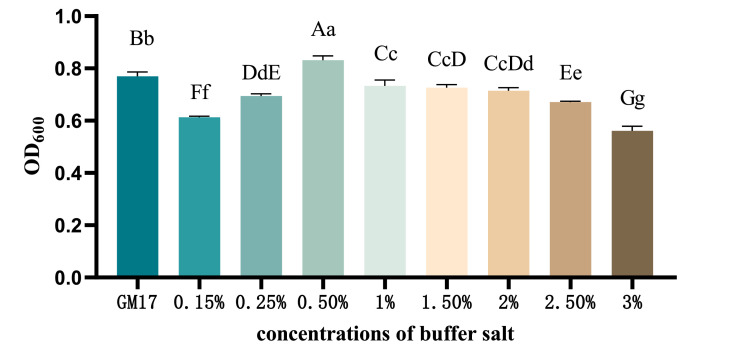
The influence of different buffer salt concentrations on the yield of RLIL4/2 strain.

Utilizing potassium dihydrogen phosphate-sodium hydroxide as buffer salts, the OD_600_ value of recombinant *ChIL-4-ChIL-2* in *Lactococcus lactis* exhibited a sequential increase across buffer salt concentrations of 0.15 %, 0.25 %, 0.5 %, and 1 %. Notably, the group with a 0.5 % concentration demonstrated a significantly higher yield compared to the other groups, as illustrated in Fig. 7.

### Response surface optimization and verification of the medium for recombinant *ChIL-4-ChIL-2* in *Lactococcus lactis*

#### Response surface optimization of the medium for recombinant *ChIL-4-ChIL-2* in *Lactococcus lactis*

A regression analysis was conducted on the experimental data presented in Table 3 utilizing Design Expert 13 software. The resulting regression equation for the response variable OD_600_ (*y_1_*) is as follows:

*y_1=_*0.657189+0.315105*x*_1_-0.0173158*x*_2_+0.610868*x*_3_-0.029*x*_1_*x*_2_+0.123368*x*_1_*x*_3_-0.00210526*x*_2_*x*_3_-0.086*x*_1_^2^+0.005*x*_2_^2^-0.435333*x*_3_^2^

**Table 3:** Results of Box-Behnken experiment.

Run	X_1_:Carbon source ratio	X_2_:Nitrogen source ratio	X_3_:Buffer salt	OD_600_
1	1	0	−1	0.920±0.006
2	1	0	1	1.099±0.004
3	0	0	0	1.056±0.006
4	1	1	0	1.054±0.017
5	1	−1	0	1.036±0.026
6	−1	0	−1	0.956±0.057
7	−1	−1	0	1.000±0.029
8	0	−1	−1	0.943±0.031
9	0	1	1	1.117±0.002
10	0	−1	1	1.075±0.003
11	0	0	0	1.057±0.022
12	0	0	0	1.061±0.009
13	−1	0	1	1.040±0.009
14	−1	1	0	1.076±0.011
15	0	1	−1	0.987±0.022

A multiple regression analysis was conducted utilizing the Design-Expert software to evaluate the regression equation. The coefficients *x*_1_, *x*_2_, *x*_3_, *x*_1_*x*_2_, *x*_1_*x*_3_, *x*_1_^2^, and *x*_3_^2^ demonstrated a high level of significance, whereas *x*_2_^2^ was found to be non-significant, and *x*_2_*x*_3_ was deemed extremely insignificant, as presented in Table 3. In cases where one or more coefficients in a significant multiple linear regression equation are identified as non-significant following significance testing, it is advisable to eliminate the corresponding independent variable with the least significance from the regression equation and to formulate a new multiple linear regression equation.

After the elimination of the *x*^2^*x*^3^ term, the optimal multiple regression equation for the response variable OD_600_ was ultimately formulated as follows:

*y_1=_*0.665478+0.315105*x*_1_-0.0185*x*_2_+0.596132*x*_3_-0.029*x*_1_*x*_2_+0.123368*x*_1_*x*_3_-0.086*x*_1_^2^+0.005*x*_2_^2^-0.435333*x*_3_^2^

The results of significance testing and variance analysis indicated that the p-value of the model was less than 0.0001, signifying that the equation model was highly significant and effectively accounted for the variability observed in the experimental data. As presented in Table 4, Table 5, the regression model demonstrated exceptional explanatory power, with a correlation coefficient (R²) of 99.84 %. This suggests that the model accurately captured the variability of the experimental data and exhibited a strong fitting effect, thereby validating the effectiveness and reliability of the experimental design. Notably, the portion of unexplained variation within the model constituted only 0.16 % of the total variation, rendering it nearly negligible. Furthermore, the lack-of-fit test results for the regression model revealed a *p*-value of 0.2562, which exceeds the significance level of 0.05. This finding further corroborates the absence of significant lack-of-fit and indicates a high degree of stability within the model. Consequently, the model is capable of performing accurate data predictions.

**Table 4:** Estimate of the partial regression coefficient of the regression equation.

**Source**	Sum of Squares	df	Mean Square	F-value	*p*-value	Significance
*x*_1_-carbon source (%)	0.0006	1	0.0006	35.99	0.0018	Sig.
*x*_2—_Nitrogen source (%)	0.0038	1	0.0038	243.18	<0.0001	Sig.
*x*_3_- buffer salt (%)	0.0346	1	0.0346	2211.73	<0.0001	Sig.
*x*_1_*x*_2_	0.0008	1	0.0008	53.78	0.0007	Sig.
*x*_1_*x*_3_	0.0023	1	0.0023	144.48	<0.0001	Sig.
*x*_2_*x*_3_	0.000003	1	0.000003	0.17	0.6986	N.S
*x*_1_²	0.0017	1	0.0017	109.15	0.0001	Sig.
*x*_2_²	0.0001	1	0.0001	5.90	0.0594	N.S
*x*_3_²	0.0104	1	0.0104	665.11	<0.0001	Sig.

**Table 5:** Regression relation analysis of variance.

**Source**	Sum of squares	df	Mean Square	F-value	*P*-value	Significance
**Model**	0.0475	9	0.0053	337.18	<0.0001	Sig.
Residual	0.0001	5	0.00			
Lack of fit	0.0001	3	0.00	3.06	0.2562	N.S
Pure error	0.0000	2	0.000007			
R^2^=0.9984						

**Fig. 8:**
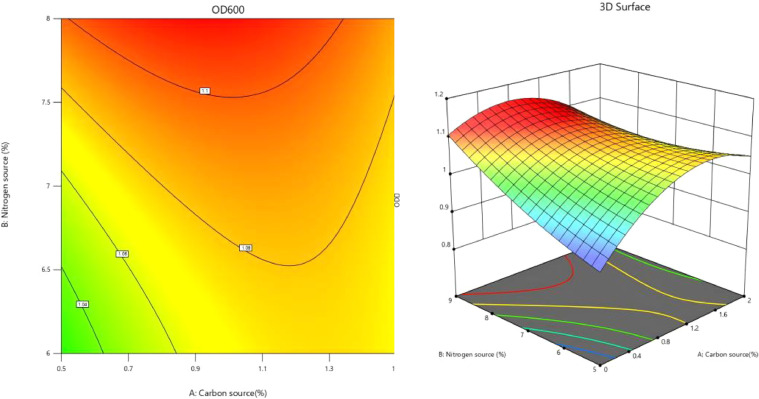
Interaction of carbon source and nitrogen source content on OD_600_ value.

**Fig. 9:**
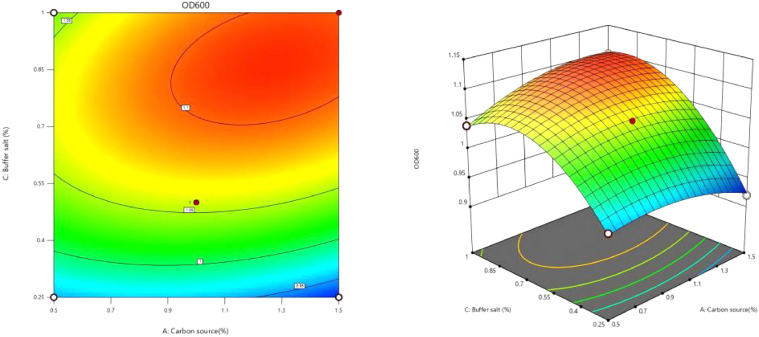
Interaction of carbon source and buffer content on OD_600_ value.

**Fig. 10:**
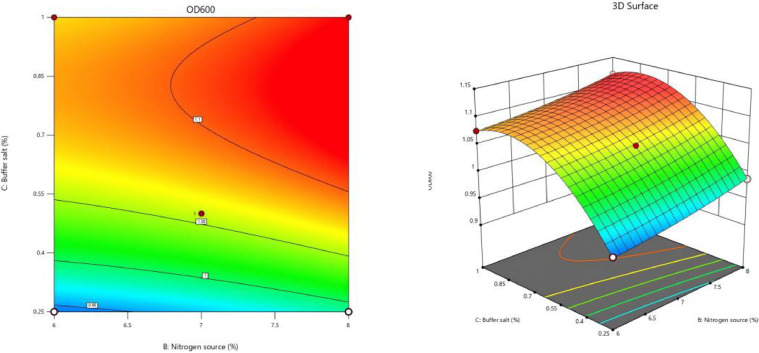
Interaction of nitrogen source and buffer salt content on OD_600_ value.

The response surface analysis plots and contour plots corresponding to the fitted regression equation were generated using software analysis, as illustrated in Fig. 8, Fig. 9, Fig. 10.

The analysis of Fig. 8 reveals that the contour plot exhibits a saddle-shaped configuration with minimal color variation. This observation suggests that the OD_600_ value of the recombinant strain is influenced by the interaction between the concentrations of carbon and nitrogen sources; however, the effect appears to be negligible. Notably, the OD_600_ value, which serves as an indicator of cell density, increases with higher percentages of both nitrogen and carbon sources. A peak in the OD_600_ value is observed when the carbon source concentration ranges from 1.1 % to 1.3 % and the nitrogen source concentration ranges from 7.5 % to 8 %. In this specific region, the contour is represented in dark red.

The data presented in Fig. 9 illustrates that the contour plot exhibits an elliptical shape, which suggests that the interaction between the carbon source content and the buffer salt content significantly influences the OD_600_ value of the recombinant strain. Specifically, when the carbon source content is maintained within the range of 0.6 % to 1.5 % and the buffer salt concentration is between 0.7 % and 1 %, a peak in the OD_600_ value of the cells is observed. This particular region is represented as a dark red ellipse on the plot.

Fig. 10 illustrates a rapid transition in the color of the contour plot from blue to red, which signifies that a steeper slope of the response surface correlates with a more pronounced interaction between the nitrogen source content and the buffer salt content on the OD_600_ value of the cells. Notably, when the nitrogen source content ranged from 7.5 % to 8 % and the buffer salt concentration varied between 0.7 % and 1 %, a peak in the OD_600_ value of the cells was observed, represented by a dark red region in the plot.

To further validate the value at the optimal point, the first-order partial derivatives of the regression equation were computed to identify the maximum value. The resulting solutions were as follows: *x*_1_ = 1.0294, *x*_2_ = 7.9987, *x*_3_ = 0.8140.

Upon substituting the independent variables into the regression equation, the resulting *y* value was 1.132. This value was subsequently converted to the actual addition amounts, which were determined to be 1 % for the carbon source, 8 % for the nitrogen source, and 0.81 % for the buffer salt. The predicted OD_600_ was also calculated to be 1.132.

#### Verification of the optimized medium for recombinant *ChIL-4-ChIL-2* in *Lactococcus lactis*

To validate the prediction results, verification experiments were conducted utilizing a carbon source composed of glucose and trehalose in a 1:1 ratio at a concentration of 1 %, a nitrogen source of yeast extract powder at 8 %, and a buffer solution consisting of potassium dihydrogen phosphate and sodium hydroxide at 0.8 %. The experiments were performed in triplicate, yielding optical density (OD_600_) values of 1.114, 1.102, and 1.094, respectively. The mean value was calculated to be 1.103, which is in close proximity to the predicted value of 1.132. Furthermore, the relative error values were all less than 5 %, thereby confirming the validity of the model, as presented in Table 6.

**Table 6:** Verification of response surface prediction results.

Run	1	2	3
OD_600_	1.114	1.102	1.094
Relative error value	1.59 %	1.01 %	1.72 %

The optimal formulation of the enrichment medium for recombinant *ChIL-4-ChIL-2* in *Lactococcus lactis* was determined based on the M17 medium through single-factor and response surface methodology. The optimal composition includes adding 1 % carbon source (a 1:1 ratio of glucose to trehalose), 8 % nitrogen source (yeast extract powder), and 0.8 % buffer salt (potassium dihydrogen phosphate combined with sodium hydroxide) to M17 medium.

## Discussion

The identification of an appropriate medium for the expression of recombinant *ChIL-4-ChIL-2* in *Lactococcus lactis* necessitates a systematic approach to experimental design and data analysis. The primary objective is to develop a method that optimizes the expression efficiency of the fusion protein, thereby facilitating further research and practical applications.

When various sugars, including trehalose, glucose, lactose, and sucrose, were utilized as substitutes for the carbon source in M17 medium, it was observed that the strain RLIL4/2 exhibited optimal growth in the presence of trehalose, followed by glucose and lactose. In contrast, sucrose did not demonstrate a significant effect on the growth of RLIL4/2. Although trehalose exhibited the most pronounced stimulatory effect on cell growth, its relatively high cost necessitates consideration of a mixture with the more economical glucose to reduce overall expenses while maintaining a comparably high cell density. Trehalose is a non-reducing disaccharide composed of two α-glucose molecules linked by a 1,1-glycosidic bond. Its physical and chemical properties are notably stable, and it does not participate in the Maillard reaction with proteins and amino acids(Paul and Paul, 2014). Research on glucosidase has indicated that following high-temperature treatment, the enzyme activity in the group supplemented with trehalose was significantly greater than that in the control group, with enhanced thermal stability attributed to the presence of trehalose(Jiang et al., 2024). Furthermore, studies have demonstrated that yeast cells accumulate substantial amounts of trehalose in response to heat shock, subsequently degrading and utilizing it immediately upon returning to normal growth temperatures, thereby engaging in active metabolic processes. The tolerance of cells to elevated environmental temperatures is positively correlated with intracellular trehalose levels(Wagner and Gasch, 2023). Numerous experiments have confirmed that trehalose, whether used as a sole carbon source or in conjunction with other carbon sources, can significantly enhance cell yield.(Zhang et al., 2021).

RLIL4/2 exhibited the most pronounced proliferation effect when utilizing yeast extract powder as the nitrogen source, demonstrating a significant advantage over other individual nitrogen sources as well as the composite nitrogen source GM17. Yeast extract powder is derived from the autolysis or enzyme-catalyzed degradation of baker's yeast or brewer's yeast and serves as a source of nitrogen, vitamins, and minerals in microbial growth and fermentation processes(Hieu et al., 2021; Zhou et al., 2022). Additionally, it is also used as a supplement in serum-free media for mammalian cell culture and human immunoglobulin. Yeast extract powder is characterized by relatively high concentrations of niacin, folic acid, and cobalamin(Jach et al., 2022) It exhibits thermal stability in neutral and acidic environments and is rich in various amino acids (Bircher et al., 2024), which significantly enhances cell growth(Da and de Assis, 2024).

In this study, we examined the differential effects of potassium dihydrogen phosphate-sodium hydroxide and β-glycerophosphate sodium as buffer salts in the RLIL4/2 and M17 medium, respectively, on cell proliferation. The findings indicated that there was no statistically significant difference in the capacity of the two buffer systems to promote cell proliferation. However, it was noted that the cost associated with potassium dihydrogen phosphate-sodium hydroxide was considerably lower than that of β-glycerophosphate sodium. The buffer system comprising potassium dihydrogen phosphate (KH_2_PO_4_) and sodium hydroxide (NaOH) demonstrates significant potential for application in chemical analysis and biochemical experiments. This system is capable of maintaining stability across a broad pH range and possesses a high buffering capacity, which effectively mitigates the influence of external acids and bases, thereby preserving the stability of the solution's pH value.(Ibrahim et al., 2013). Furthermore, given that phosphate is a naturally occurring component in biological cells, this buffer system exhibits excellent biocompatibility, making it suitable for a wide array of biochemical experiments. In light of the aforementioned analysis, the use of potassium dihydrogen phosphate-sodium hydroxide as the buffer salt in the RLIL4/2 medium not only facilitates cell proliferation but also significantly reduces the overall cost of the medium.

## Conclusion

The optimal enrichment medium for the production of recombinant *ChIL-4-ChIL-2* in *Lactococcus lactis* was identified as follows: a carbon source comprising a 1:1 ratio of glucose to trehalose at a concentration of 1 %, a nitrogen source comprising 8 % yeast extract powder, a buffer system utilizing potassium dihydrogen phosphate and sodium hydroxide at a concentration of 0.8 %, sodium ascorbate at 0.05 %, and magnesium sulfate at 0.025 %, all added to M17 medium. When recombinant *ChIL-4-ChIL-2* in *Lactococcus lactis* was cultured in this medium in an Erlenmeyer flask at a temperature of 30°C under static conditions, the cell density achieved was 1.103.

## Declaration of competing interest

Haoyu-Zhang carried out most of the experiments, wrote the manuscript, and should be considered as primary author. Ming-xue Zheng critically revised the manuscript and the experiment design. Yu-tong Yang, Xue-qi Zhang, Si-chen Dong, Li-wen Wang, Ying-ying Chen, Li Zhang, Xiao-ling Lv, Rui Bai helped with the experiment. All the authors read and approved the final version of the manuscript.
